# EEG and Sleep Effects of Tramadol Suggest Potential Antidepressant Effects with Different Mechanisms of Action

**DOI:** 10.3390/ph14050431

**Published:** 2021-05-04

**Authors:** Szabolcs Koncz, Noémi Papp, Noémi Menczelesz, Dóra Pothorszki, György Bagdy

**Affiliations:** 1 Department of Pharmacodynamics, Semmelweis University, 1085 Budapest, Hungary; koncz.szabolcs@pharma.semmelweis-univ.hu (S.K.); papp.noemi@pharma.semmelweis-univ.hu (N.P.); menczelesz.noemi@pharma.semmelweis-univ.hu (N.M.); dorapothorszki@gmail.com (D.P.); 2 MTA-SE Neuropsychopharmacology and Neurochemistry Research Group, Hungarian Academy of Sciences, Semmelweis University, 1085 Budapest, Hungary; 3 NAP-2-SE New Antidepressant Target Research Group, Hungarian Brain Research Program, Semmelweis University, 1085 Budapest, Hungary

**Keywords:** tramadol, sleep, depression, antidepressant, pharmaco-EEG, brain oscillation, EEG power spectra, rat, sleep-wake cycle, chronic pain

## Abstract

Tramadol is a widely used, centrally acting, opioid analgesic compound, with additional inhibitory effects on the synaptic reuptake of serotonin and noradrenaline, as well as on the 5-HT_2_ and NMDA receptors. Preclinical and clinical evidence also suggests its therapeutic potential in the treatment of depression and anxiety. The effects of most widely used antidepressants on sleep and quantitative electroencephalogram (qEEG) are well characterized; however, such studies of tramadol are scarce. Our aim was to characterize the effects of tramadol on sleep architecture and qEEG in different sleep–wake stages. EEG-equipped Wistar rats were treated with tramadol (0, 5, 15 and 45 mg/kg) at the beginning of the passive phase, and EEG, electromyogram and motor activity were recorded. Tramadol dose-dependently reduced the time spent in rapid eye movement (REM) sleep and increased the REM onset latency. Lower doses of tramadol had wake-promoting effects in the first hours, while 45 mg/kg of tramadol promoted sleep first, but induced wakefulness thereafter. During non-REM sleep, tramadol (15 and 45 mg/kg) increased delta and decreased alpha power, while all doses increased gamma power. In conclusion, the sleep-related and qEEG effects of tramadol suggest antidepressant-like properties, including specific beneficial effects in selected patient groups, and raise the possibility of a faster acting antidepressant action.

## 1. Introduction

Tramadol (1*RS*,2*RS*)-2-[(dimethylamino)-methyl]-1-(3-methoxyphenyl)-cyclohexanol] hydrochloride is a widely used, centrally acting opioid analgesic in the treatment of acute and chronic pain [1]. Tramadol acts as a weak agonist on µ-opioid receptors and inhibits serotonin (5-HT) and noradrenaline (NA) reuptake. Furthermore, it shows antagonist properties on 5-HT_2_, muscarinic acetylcholine, as well as NMDA receptors [2]. Tramadol is considered a weak opioid receptor agonist, but the analgesic effects of tramadol are also mediated through its main metabolite, O-desmethyltramadol, which has a greater affinity to µ-opioid receptors and has a more potent analgesic effect [3,4].

Given the monoaminergic effects of tramadol, which is characteristic of most antidepressants, and its structural similarities to venlafaxine (a reuptake inhibitor antidepressant), several preclinical studies have investigated the potential antidepressant effects of tramadol [5,6,7]. Early preclinical studies have reported the antidepressant effects of tramadol in rodents [8,9]. Using a novel network-based drug repositioning method, Zhang et al. have proposed the antidepressant-like effects of tramadol [10]. Recently, machine learning analysis applied on patient drug reviews on WebMD predicted the repurposing indication of tramadol in the treatment of depression [11]. Moreover, another database mining on patient drug reviews suggested that tramadol has a fast-onset antidepressant effect [12].

A major depressive disorder often co-occurs with sleep disruption. The vast majority of patients with depression suffer from impaired sleep, such as insomnia or hypersomnia [13]. The latter symptoms define the type of antidepressant used in these subgroups of patients [14]. Characteristic sleep-EEG alterations in patients with depression include disinhibition of rapid eye movement (REM) sleep, disruption of sleep continuity, and changes in non-rapid eye movement (NREM) sleep. Additionally, several depressed patients show reduced electroencephalogram (EEG) delta power throughout the night [13].

Most antidepressants show characteristic effects on sleep, both in healthy volunteers and depressed patients. Reuptake and monoamine-oxidase (MAO) inhibitor antidepressants increase REM sleep latency time and suppress REM sleep time, both in humans and animals [15,16]. Some other effects on sleep-EEG or EEG variables may be suitable as biomarkers for the prediction of therapy response in depression [17,18]. Therefore, the sleep-EEG related effects of investigational and clinically used antidepressants are extensively studied in humans and animals [13,14,19].

Tramadol is considered to have great potential in the treatment of depression beyond its analgesic effect; however, only limited information is available regarding its sleep-related effects. Only one relevant study could be found that has reported a sleep-disturbing effect in eight healthy volunteers when applying tramadol in a single dose (50 or 100 mg) during the night following its application. Furthermore, a 100 mg dose of tramadol shortened the duration of REM sleep, suggesting that the latter effect is dose-dependent, although clinically relevant higher doses were not tested [20]. Therefore, in this study, we investigated the dose-dependent effects of acutely administered tramadol on rat sleep architecture and EEG power. Our results confirm the potential antidepressant effects of tramadol and suggest beneficial effects for treating sleep disturbance in specific subgroups of depressed patients.

## 2. Results

Tramadol, given at the beginning of the passive phase, i.e., light phase of the dark/light cycle induced dose-dependent effects on sleep and wake parameters and qEEG.

### 2.1. Effects of Tramadol on the Pattern of Sleep–Wake Cycle

#### 2.1.1. Effects on Wakefulness

**Figure 1:**
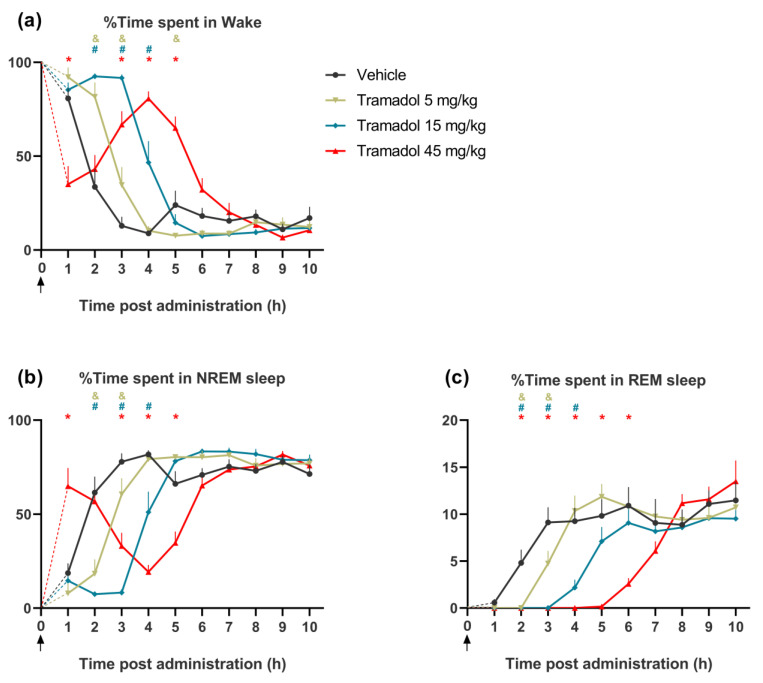
The effects of intraperitoneally (i.p.) administered single doses of tramadol (5, 15 and 45 mg/kg) or vehicle on the amount of (**a**) wakefulness, (**b**) non-rapid eye movement (NREM) sleep and (**c**) rapid eye movement (REM) sleep for 10 h after the injections. The arrows show the time of the administration, at the beginning of the passive phase. At this time point, all animals were awake. The mean value of the first hour is connected with time zero by dashed lines. The &, # and * signs represent significant results (*p* < 0.05) of the post hoc tests, compared to vehicle in the case of the 5, 15 and 45 mg/kg tramadol-treated groups, respectively. Data are presented as mean ± SEM of *n* = 8 rats/group.

Tramadol dose-dependently affected time spent in wakefulness and this effect changed during the 6 h, post-injection (treatment: *F*_(3, 21)_ = 21.95, *p* < 0.0001, treatment × time interaction: *F*_(27, 189)_ = 22.36, *p* < 0.0001, Figure 1a). Lower doses of the drug (5 and 15 mg/kg) promoted wakefulness during the first 2–4 h, while the highest dose (45 mg/kg) first decreased (1st hour), and then, at 3–5 h, increased the amount of wakefulness.

#### 2.1.2. Effects on NREM Sleep

In parallel with its effects on wakefulness, tramadol dose-dependently affected the time spent in NREM sleep (treatment: *F*_(3, 21)_ = 11.03, *p* = 0.0001, treatment × time interaction: *F*_(27, 189)_ = 21.73, *p* < 0.0001, Figure 1b). Lower doses of tramadol (5 and 15 mg/kg) reduced time spent in NREM sleep during the first 2–4 h, while the highest dose of tramadol (45 mg/kg) increased NREM sleep time in the first hour, and decreased NREM sleep during 3–5 h.

#### 2.1.3. Effects on REM Sleep

**Figure 2:**
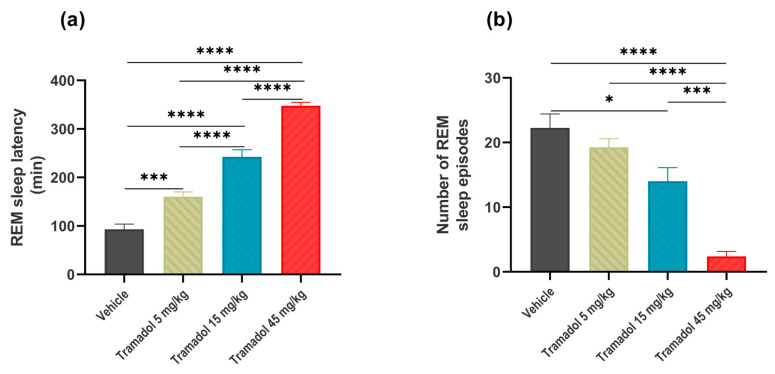
The effects of intraperitoneally administered single doses of tramadol (5, 15 and 45 mg/kg i.p.) or vehicle on (**a**) REM sleep latency following the first non-rapid eye movement (NREM) sleep episode and (**b**) the amount of rapid eye movement (REM) sleep during the first 6 h after drug administration. Significant post hoc results are marked by * *p* < 0.05, *** *p* < 0.001, **** *p* < 0.0001. Data are presented as mean ± SEM (*n* = 8 rats/group).

The time spent in REM sleep was also dose-dependently reduced after tramadol administration, but, interestingly, the effect of the highest dose now was not opposite to those with lower doses at the onset of the effect (treatment: *F*_(3, 21)_ = 14.31, *p* < 0.0001, treatment × time interact: *F*_(27, 189)_ = 5.613, *p* < 0.0001, Figure 1c). We also found dose-dependent decreases in the REM sleep latency (*F*_(3, 21)_ = 112.1, *p* < 0.0001, Figure 2a), and the number of REM sleep episodes (*F*_(3, 21)_ = 24.47, *p* < 0.0001, Figure 2b).

### 2.2. Effects of Tramadol on qEEG

**Figure 3:**
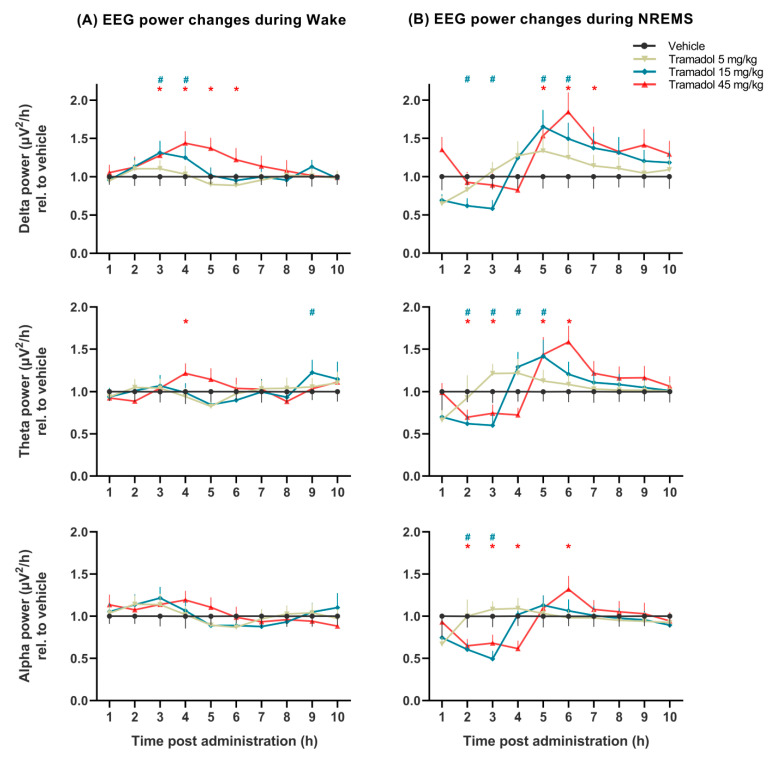
Delta, theta, and alpha EEG power changes induced by tramadol (5, 15 and 45 mg/kg i.p.) during (**A**) wakefulness, and (**B**) non-rapid eye movement sleep (NREMS) with the calculated SEM for each hour. EEG power data were averaged for each hour after administration of vehicle or tramadol at the beginning of the passive phase. The # and * signs indicate significant post hoc results (*p* < 0.05) compared to vehicle in case of the 15 and 45 mg/kg tramadol-treated groups, respectively. Data are presented as SEM (*n* = 8 rats/group).

**Figure 4:**
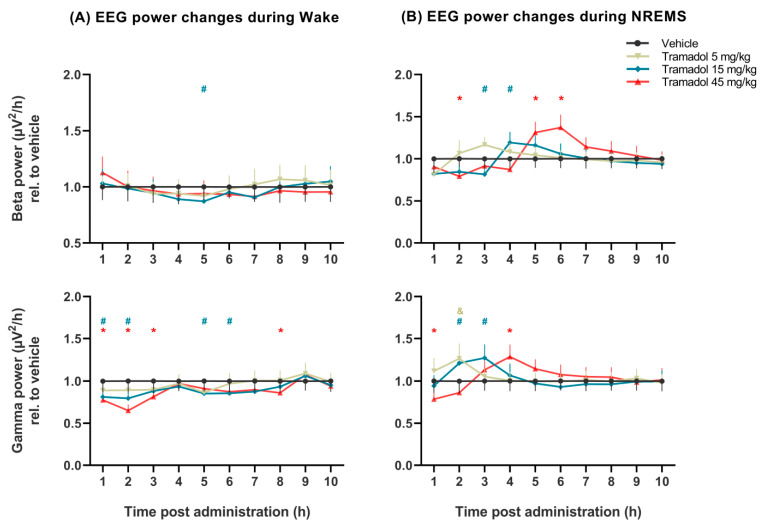
Beta and gamma EEG power changes induced by tramadol (5, 15 and 45 mg/kg i.p.) during (**A**) wakefulness and (**B**) non-rapid eye movement sleep (NREMS) compared to vehicle (presented as value 1.0 with the calculated SEM for each hour). EEG power data were averaged for each hour after administration of vehicle or tramadol at the beginning of the passive phase. The &, # and * signs indicate significant post hoc results (*p* < 0.05) compared to vehicle in case of the 5, 15 and 45 mg/kg tramadol-treated groups, respectively. Data are presented as SEM (*n* = 8 rats/group).

The effects of tramadol on qEEG were much more prominent in NREM sleep than in wakefulness (Figure 3 and Figure 4).

Delta power during NREM sleep was markedly increased by the two higher doses 5 h after administration (treatment: *F*_(3, 21)_ = 11.28, *p* = 0.0001, treatment × time interaction: *F*_(27, 184)_ = 5.570, *p* < 0.0001, Figure 3B). Notably, the 15 mg/kg of tramadol beforehand decreased delta power transiently. Delta power during wakefulness was marginally increased by tramadol (15 and 45 mg/kg) 3 h after administration (treatment: *F*_(3, 21)_ = 16.51, *p* < 0.0001, treatment × time interaction: *F*_(27, 189)_ = 4.913, *p* < 0.0001, Figure 3A).

Theta power was altered in NREM sleep, namely, a transient decrease followed by an increase was observed after the two higher doses (no significant treatment effect: *F*_(3, 21)_ = 1.352, *p* = 0.2846, significant treatment × time interaction: *F*_(27, 184)_ = 6.372, *p* < 0.0001, Figure 3B).

Alpha power was significantly decreased during NREM sleep by tramadol at doses of 15 and 45 mg/kg (treatment: *F*_(3, 21)_ = 3.526, *p* < 0.05, treatment × time interaction: *F*_(27, 184)_ = 7.198, *p* < 0.0001, Figure 3B), while during wakefulness tramadol showed no effect on alpha power (treatment: *F*_(3, 21)_ = 0.4208, *p* = 0.7400, Figure 3A).

Beta power was slightly transiently affected by the two higher doses of tramadol in NREM sleep (no significant treatment effect: *F*_(3, 21)_ = 1.745, *p* = 0.1886, significant treatment × time interaction: *F*_(27, 184)_ = 6.914, *p* < 0.0001, Figure 4B). This effect was a decrease first, followed by an increase in the case of both higher doses, although it was shifted in time.

Gamma power was significantly increased by all doses of tramadol during NREM sleep (treatment × time interaction: *F*_(27, 184)_ = 5.347, *p* < 0.0001), although the treatment effect alone did not reach significance level (Figure 4B). In contrast, tramadol slightly reduced gamma power during wakefulness (treatment: *F*_(3, 21)_ = 12.89, *p* < 0.0001, treatment × time interaction: *F*_(27, 189)_ = 2.596, *p* < 0.0001; Figure 4A).

The effects of different doses of the drug on the EEG powers at specific hours (results of Bonferroni post hoc comparisons) are shown in Figure 3 and Figure 4.

## 3. Discussion

Here, we report that acute tramadol treatment markedly affects the architecture of the sleep–wake cycle and modulates qEEG in a sleep–wake stage dependent manner in Wistar rats.

With regard to the pattern of sleep–wake stages, the most prominent effect of tramadol was found on REM sleep. Namely, tramadol dose-dependently reduced both the duration of REM sleep and the number of REM episodes, as well as increased REM sleep latency. These effects of tramadol are in line with earlier data in humans [20] and with the REM suppressing effects of reuptake inhibitor antidepressants, such as selective serotonin reuptake inhibitors (SSRIs), serotonin and noradrenaline inhibitors (SNRIs), and tricyclic antidepressants (TCAs), both in humans and in laboratory animals [19,21,22]. Alterations in REM sleep, such as shortened REM sleep onset and increased REM sleep duration, have been linked to depressive states, and have also been considered as biomarkers of depression. Almost all reuptake inhibitor antidepressants markedly suppress REM sleep, which is thought to be an important component of their therapeutic effect [15].

Serotoninergic and noradrenergic neurotransmissions play an important role in the regulation of REM sleep; mainly monoaminergic neurons in the dorsal raphe and locus coeruleus activate the REM-off circuitry and suppress REM sleep [23,24,25]. Earlier in vitro studies have demonstrated the inhibitory effects of tramadol on 5-HT and NA reuptake in the dorsal raphe nucleus and in the locus coeruleus, respectively [26,27]. Indeed, in vivo microdialysis experiments performed on freely moving rats have shown that tramadol increased extracellular 5-HT and NA levels in the ventral hippocampus. In analgesic doses, the effects of tramadol on monoamine levels were comparable to dual reuptake inhibitors, such as duloxetine, venlafaxine, and clomipramine [28]. Thus, the suppressive effect of tramadol on REM sleep is most likely mediated by its inhibitory effects on the reuptake of 5-HT and NA, but complementary mechanisms might also play a role.

Besides its opioid agonist and reuptake inhibitory effects, tramadol exerts inhibitory effects on several receptor types, such as muscarinic acetylcholine receptors, NMDA receptors, and 5-HT_2_ receptors [2]. Importantly, the anticholinergic scopolamine, the NMDA antagonist ketamine, and the 5-HT_2C_ receptor antagonist SB-242084 all have fast-onset antidepressant properties [29,30], and also markedly suppress REM sleep [31,32], suggesting that the effect of tramadol on muscarinic, NMDA and 5-HT_2_ receptors may be key in its antidepressant effect. Furthermore, data mining on the patient drug reviews database has also suggested that tramadol has a fast-onset antidepressant effect [12]. Taken together, these data raise the possibility of tramadol’s fast-onset antidepressant properties.

Tramadol dose-dependently affected time spent in wakefulness and NREM sleep; namely, lower doses promoted wakefulness, while the highest dose first decreased, and then increased the amount of wakefulness. The wakefulness promoting effects of tramadol are in line with the results of an exploratory qualitative study, where participants described tramadol as an effective drug for relieving fatigue or tiredness and stress [33]. Our data supports this notion, thus tramadol is more likely to be effective in depressed patients with hypersomnia, or at least in patients who do not have problems falling asleep. In patients who have insomnia, tramadol might be used with caution.

Tramadol markedly increased delta EEG power during NREM sleep and marginally in wakefulness. Delta activity is typically associated with NREM sleep states and has also been linked with cognitive process and motivation during wakefulness [34]. Chronic exposure to stress in rats decreased delta power during NREM sleep [35]. In line with this, patients suffering from major depression also show reduced EEG delta power throughout sleep [13]. Regarding the effect of antidepressants on delta EEG power, we showed previously that acute escitalopram (SSRI) treatment increased delta power during active wakefulness in rats [36]. Moreover, antidepressants with 5-HT_2_ antagonist properties (e.g., mirtazapine, mianserin and trazodone) mainly increased slow wave sleep and sleep efficiency, without any significant effects on REM sleep [14,37]. This slow wave sleep enhancing effect might be mediated by 5-HT_2A_ and 5-HT_2C_ receptors, as previous studies show that non-selective 5-HT_2_ receptor antagonist ritanserin also increased delta activity and slow wave sleep [38]. Ogata et al. showed that tramadol in pharmacologically relevant concentrations competitively inhibits the effects of 5-HT on 5-HT_2C_ receptors [39]. Not only the main compound, but the primary metabolite of tramadol, O-desmethyltramadol, also has inhibitory effects on 5-HT_2C_ receptors [40]. Therefore, tramadol’s long-term effects on delta oscillations might be explained by its antagonist effects on 5-HT_2C_ receptors, although the role of its antagonistic effects on 5-HT_2A_ receptors cannot be ruled out.

The nonselective NMDA receptor antagonist ketamine, which is known to evoke an immediate antidepressant effect, also increased the EEG delta power during NREM sleep in rats when applied in single doses [41]. This effect also seems to be important in clinical settings, because low baseline delta sleep predicted a response to ketamine in patients with treatment-resistant major depressive disorders [42]. Since tramadol also has inhibitory effects on NMDA receptors, involvement of this mechanism in the antidepressant-like effects of tramadol has also been proposed [43,44]. Indeed, tramadol pretreatment before ketamine administration elicited greater antidepressant effects in the rat forced swimming test. Moreover, the co-administration of sub-effective doses of tramadol and ketamine together elicited antidepressant-like effects in the mouse forced swimming test [44,45]. Overall, tramadol’s effect on delta oscillation might be mediated by its activity on 5-HT_2C_ and NMDA receptors. Anyway, tramadol’s delta activity enhancing effect during NREM sleep could be beneficial, especially in patients with a decreased delta sleep ratio.

During wakefulness, tramadol had no effect in any of the applied doses on alpha power. However, during NREM sleep, tramadol significantly decreased alpha power at the doses of 15 and 45 mg/kg. In mammals, delta oscillations dominate the EEG during NREM sleep, and alpha oscillations are decreased [34]. On the other hand, alpha wave intrusion in delta sleep has been observed in patients with major depression and patients with fibromyalgia [46,47]. Fibromyalgia is associated with chronic and diffuse musculoskeletal pain, which often co-occurs with sleep disturbances and mood changes [48]. In healthy volunteers, artificially induced alpha activity during NREM sleep produces fibromyalgia-like symptoms; therefore, increased alpha activity may even be the source of these symptoms [49]. The alpha EEG power reducing effects of tramadol during NREM sleep reported in our study are in line with recent clinical evidence that suggests the positive effect of tramadol on the symptoms of fibromyalgia [48].

Acute tramadol slightly decreased gamma power during wakefulness, while it was elevated during NREM sleep. Gamma EEG power has gained interest recently as a biomarker for depression. Moreover, it has also been suggested that gamma oscillation may provide information about the therapeutic effects of all antidepressants [50,51]. In contrast to tramadol, ketamine and its metabolite (2*R*,6*R*)-hydroxynorketamine, in effective antidepressant doses, have been shown to increase gamma power in mice during wakefulness [52]. However, acute treatment with antidepressants such as fluoxetine and citalopram suppress gamma power in rats, similarly to tramadol [53,54]. Gamma suppression might be the result of elevated 5-HT levels, which is further proved by the fact that an evoked 5-HT release, through the electrical stimulation of the dorsal raphe nucleus in rats, also decreased gamma power [55]. Gamma oscillation has been associated with perceptual and higher cognitive processes in healthy human subjects and animals [56]. However, abnormally high gamma power during wakefulness has been associated with positive symptoms of schizophrenia, such as hallucinations [57]. During sleep, beta and gamma oscillation contribute to the reactivation of information, and therefore may have an important role in memory consolidation [58]. Ketamine and certain other NMDA receptor antagonists increased gamma power during NREM sleep similarly to tramadol [59]. Previously, we showed that chronic, but not acute, antidepressant treatment elicited an increase in gamma power during NREM sleep [36]. Additionally, the acute administration of SB-242084, a 5-HT_2C_ receptor antagonist with fast-onset antidepressant-like properties, also elevated gamma power in slow wave sleep [30,60]. Therefore, tramadol’s acute effects on gamma oscillation during NREM sleep might be mediated by its antagonist effects on 5-HT_2C_ receptors or NDMA receptors, and may suggest a faster-onset antidepressant-like action. Indeed, clinical observations and data mining analysis support the rapid onset antidepressant properties of tramadol [12,61].

Tramadol is a racemic compound and consists of two enantiomers with different pharmacological effects. Namely, (+)-tramadol is mainly responsible for 5-HT reuptake inhibition and µ-opioid agonism, while (−)-tramadol mediates the NA reuptake inhibitor effects of racemic tramadol [62,63]. We evaluated only the effects of the racemic compound because, in clinical settings, only the racemic tramadol is in use. Future work may discover enantiomer-specific effects of tramadol on sleep and EEG. Additionally, tramadol is often used long-term in the management of pain or in off-label treatment of psychiatric conditions [64], underlining the importance of future investigations into the effects of chronically administered tramadol on vigilance and qEEG.

In conclusion, our findings provide evidence that acute tramadol markedly affects sleep parameters, also including those sleep parameters that can be crucial in the pathophysiology of depression. The REM-suppressing effects of tramadol are similar to those of 5-HT and NA reuptake blocker antidepressants, while its delta power-inducing effects during NREM sleep resemble those of 5-HT receptor antagonist antidepressants. Some qEEG effects of tramadol are similar to those observed with chronic administration of reuptake blockers or the acute effects of fast acting antidepressants. Effects on alpha power suggest possible therapeutic value in fibromyalgia. Thus, the sleep related and qEEG effects of tramadol suggest antidepressant-like properties and raise the possibility of faster acting antidepressant action, with specific beneficial effects in selected patient groups. Furthermore, these data support the possible use of tramadol in fibromyalgia.

## 4. Materials and Methods

### 4.1. Animals

All animal experiments and housing conditions were conducted in accordance with the EU Directive 2010/63/EU and specific national laws (the Hungarian Governmental Regulations on animal studies 40/2013). All efforts were made to minimize the number of animals, as well as their pain and discomfort.

Male Wistar rats (Han:WIST, Toxi-Coop, Hungary) were used in the experiments. The animals were maintained under controlled environmental conditions (21 ± 1 °C temperature, 12-h/12-h light/dark cycle with light on at 10 a.m.). Water and standard rodent food were available ad libitum.

### 4.2. Surgery

All rats were implanted with electroencephalographic (EEG) and electromyographic (EMG) electrodes, as described earlier [65], under 2% isoflurane anesthesia. In brief, for fronto-parietal EEG recordings, stainless steel screw electrodes were placed epidurally over the left frontal cortex (1.5 mm lateral and 2.0 mm anterior to bregma), left parietal cortex (1.5 mm lateral and 2.0 mm anterior to lambda), and over the cerebellum as a ground electrode. For EMG recordings, a pair of EMG electrodes (stainless steel spring electrodes covered by silicon rubber, Plastics One Inc., Roanoke, VA, USA) was placed into the neck musculature. The rats weighed 300–330 g at surgery.

After a recovery period (8–10 days), the rats were kept individually in glass recording chambers, attached to the EEG system by a recording cable. An electric swivel fixed above the cages permitted free movement of the rats. The animals remained connected throughout the whole study.

### 4.3. Drugs

Tramadol hydrochloride was purchased from Sigma-Aldrich (42965-5G-F, chemical purity ≥99%). The animals were treated intraperitoneally (i.p.) with 5, 15 or 45 mg/kg of tramadol or vehicle (saline 1 mL/kg), at the beginning of the light phase, just before the recordings. Doses of tramadol were chosen on the basis of depression related behavioral rat studies [66,67] and a previous in vivo microdialysis experiment [28]. All rats received all treatments in a randomized crossover design with a 5-day-long washout period between the treatments.

### 4.4. EEG Recording and Analysis

EEG, EMG, and motor activity were recorded for 10 h after each treatment (Coulburn Lablinc System, Holliston, MA, USA). The animals remained undisturbed throughout the recordings. The signals were amplified (EEG: 5000 times, EMG: 10,000 times) and filtered (below 0.50 Hz, above 100 Hz). Analog-to-digital conversion was performed at 256 Hz sampling rate.

Sleep–wake stages were differentiated in 4-s epochs, using conventional criteria [65], as follows: in wakefulness, the EEG was characterized by low-amplitude activity at beta (14–29 Hz) and alpha (10–13 Hz) frequencies, accompanied by high EMG and motor activity. In NREM sleep, the EEG was characterized by high-amplitude activity in the delta (0.5–4 Hz) frequency band, sometimes interrupted by spindles (6–15 Hz), accompanied by reduced EMG activity and minimal motor activity. In REM sleep, the EEG was characterized by low-amplitude, high-frequency activity, and regular theta waves (5–9 Hz), accompanied by neither EMG nor motor activity, except for occasional twitching. For scoring the vigilance stages, we first used the automatic scoring function of Sleep Sign for Animal software (Kissei Comtec America Inc., Fort Lee, NJ, USA); then, visual supervision was carried out by researchers who were blind to the treatment of the rats. Epochs containing artefacts or stage transitions were excluded from the power spectral analysis.

The following sleep–wake parameters were calculated: time spent in wakefulness, NREM sleep, and REM sleep in each hour; number of REM sleep episodes in the first half of the passive phase; and REM sleep latency. REM sleep latency was calculated as the time elapsed between first NREM sleep episode and the beginning of the first consecutive REM sleep episode lasting at least 7 epochs.

EEG power spectral analysis was performed at the frequency range of 0.50–60 Hz (fast Fourier transformation, Hanning window, frequency resolution: 0.25 Hz). The 0.25-Hz bins were summed into 1-Hz bins, marked by their upper limits. Bins above 60 Hz were excluded. Power values were averaged hourly, in wakefulness, NREM sleep, and REM sleep. Then, the power values of each hour were averaged in the delta (1–4 Hz), theta (5–9 Hz), alpha (10–13 Hz), beta (14–29 Hz), and gamma (30–60 Hz, excluding 49–51 Hz) frequency bands.

### 4.5. Statistics

To evaluate the effect of different doses of tramadol on the time spent in wakefulness, NREM sleep, and REM sleep for each hour, a two-way double repeated measure ANOVA was used (treatment and time, both as repeated variables). For the analysis of the effects of tramadol on the amount of REM sleep and REM sleep latency, a repeated measure one-way ANOVA was performed. For qEEG data analyses, a mixed-model design ANOVA (treatment and time, both as repeated variables) was used. For multiple comparisons, a Bonferroni post hoc test was performed.

## Data Availability

The datasets generated and analyzed during the current study are not publicly available due to ongoing analysis for future publication, but are available from the corresponding author upon reasonable request.
